# Granulocyte colony-stimulating factor-producing esophageal squamous cell carcinoma following chemoradiotherapy and bone marrow transplantation for acute lymphoblastic leukemia

**DOI:** 10.1007/s10388-013-0387-3

**Published:** 2013-08-28

**Authors:** Shuhei Mayanagi, Masahiro Niihara, Hironobu Goto, Tomoya Yokota, Hiroyuki Tabuse, Hiroshi Yasui, Hirofumi Ogawa, Tetsuo Nishimura, Kimihide Kusafuka, Yasuhiro Tsubosa

**Affiliations:** 1 Division of Esophageal Surgery, Shizuoka Cancer Center Hospital, 1007 Shimonagakubo, Nagaizumi-cho, Sunto-gun, Shizuoka, 411-8777 Japan; 2 Division of Gastric Surgery, Shizuoka Cancer Center Hospital, Shizuoka, Japan; 3 Division of Gastrointestinal Oncology, Shizuoka Cancer Center Hospital, Shizuoka, Japan; 4 Division of Radiation Oncology, Shizuoka Cancer Center Hospital, Shizuoka, Japan; 5 Division of Pathology, Shizuoka Cancer Center Hospital, Shizuoka, Japan

**Keywords:** Esophageal squamous cell carcinoma, Granulocyte colony-stimulating factor, Second primary cancer, Radiation-induced esophageal cancer, Bone marrow transplantation

## Abstract

A 30-year-old man, who had been treated with craniospinal irradiation, total-body irradiation, and bone marrow transplantation for acute lymphoblastic leukemia at 20 years of age, complained of dysphagia. The patient had spike fever with leukocytosis (19,020/μl). Serum granulocyte colony-stimulating factor (G-CSF) level was also increased (53.7 pg/ml). Immunohistochemistry revealed positive staining for anti-G-CSF antibody in carcinoma cells obtained by endoscopic biopsy. The patient was diagnosed with G-CSF-producing locally advanced esophageal squamous cell carcinoma. The clinical diagnosis was T4; tumor invaded aorta, with regional lymph node metastases (N1). The patient underwent transthoracic esophagectomy with three-field lymph node dissection and gastric tube reconstruction following a radiation dose of 41.4 Gy with 5-fluorouracil continuous infusion as neoadjuvant therapy. There were no viable cancer cells in the resected esophageal specimen and lymph nodes. The patient had no evidence for typical risk factors for developing esophageal cancer. After the operation, neutrophils and G-CSF decreased to normal levels. The patient had recurrence of regional and distant multiple lymph node metastases at 3 months after operation.

## Introduction

New treatments for childhood cancer have decreased the number of deaths from the primary cancer. However, the treatment of cancer—including chemotherapy, radiation therapy or stem cell transplantation—may cause various late effects for childhood cancer survivors months or years after successful treatment has ended. Large cohort studies demonstrated increasing risk of second and subsequent primary cancer [1–3]. One of the most common causes of death in childhood cancer survivors is second primary cancer forms. Solid tumors may appear more than 10 years after primary cancer diagnosis and treatment. When radiation therapy and chemotherapy are given together, the risk of gastrointestinal neoplasm is even higher [4]. The second esophageal cancer might occur within the irradiated field of the mediastinal or spinal region.

Meanwhile, granulocyte colony-stimulating factor (G-CSF)-producing malignant tumor with leukocytosis has been reported to occur in various organs. G-CSF-producing tumor was first described by Robinson [5], and has been described in lung cancer [6] and gastroenterological tumors [7–9]. There are few reports on G-CSF-producing esophageal cancer, and most of them have been associated with poor clinical outcomes.

We report herein a rare case of esophageal squamous cell carcinoma following therapy for acute lymphoblastic leukemia that showed increased leukocyte and serum G-CSF levels. Expression of G-CSF in the cytoplasm of cancer cells was found by immunohistochemical staining.

### Case report

**Fig. 1:**
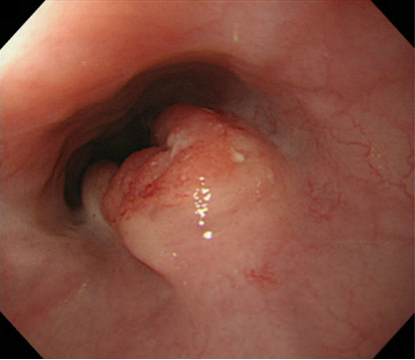
Esophagogastroduodenoscopy showed a tumor with ulcerating lesions surrounded by elevated borders in the upper esophagus, 25–34 cm from incisors

**Fig. 2:**
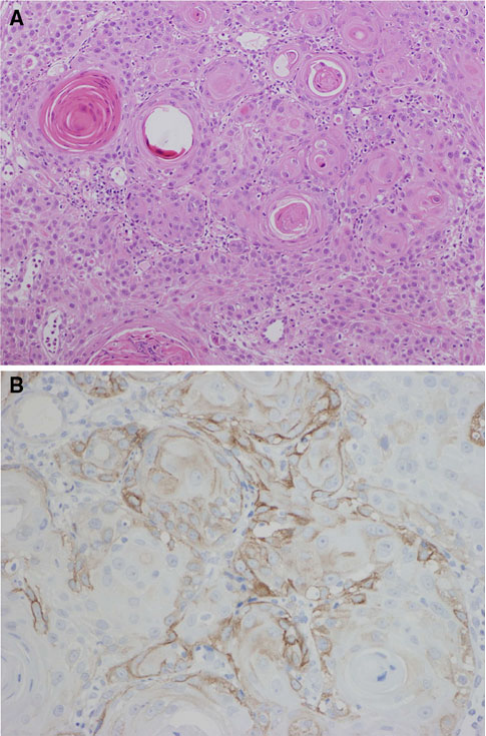
**a**, **b** Histological findings for the endoscopic biopsied specimen. Well-differentiated squamous cell carcinoma with intercellular bridge and keratin pearl formation was seen on hematoxylin and eosin staining (**a**, ×100). Immunohistochemistry revealed positive staining for granulocyte colony-stimulating factor (G-CSF) in the cytoplasm of squamous cell carcinoma (**b**)

A 30-year-old man who had history of treatment for acute lymphoblastic leukemia complained of dysphagia. He had received initial induction chemotherapy, including l-asparaginase, vincristine, and steroid, and whole-brain radiotherapy of 18 Gy at age 11 years. At age 14 years, radiation to the whole brain of 24 Gy and the whole spine of 18 Gy were performed for central nervous system recurrence. At age 20 years, because of bone marrow relapse, he was treated with remission reinduction chemotherapy, bone marrow transplantation, and total-body irradiation of 12 Gy. Thus, the esophagus might have been exposed to radiation of 30 Gy during the overall treatment for acute lymphoblastic leukemia. Esophagogastroduodenoscopy showed a type 2 advanced cancer in the upper thoracic esophagus (Fig. 1). The specimens taken by endoscopic biopsy were histologically proven to be well-differentiated squamous cell carcinoma (Fig. 2a).

**Fig. 3:**
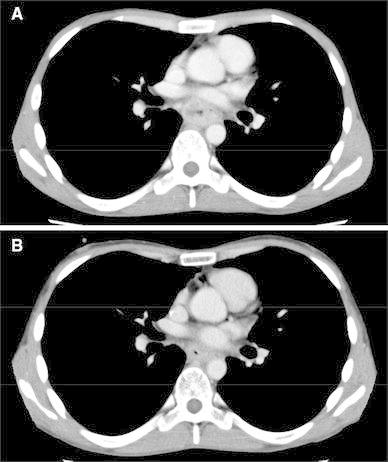
Preoperative radiological images. Computed tomography (CT) scans revealed the tumor invasion of surrounding tissue including aorta prior to initiating neoadjuvant chemoradiotherapy (**a**). The tumor decreased in size after neoadjuvant chemoradiotherapy (**b**)

**Fig. 4:**
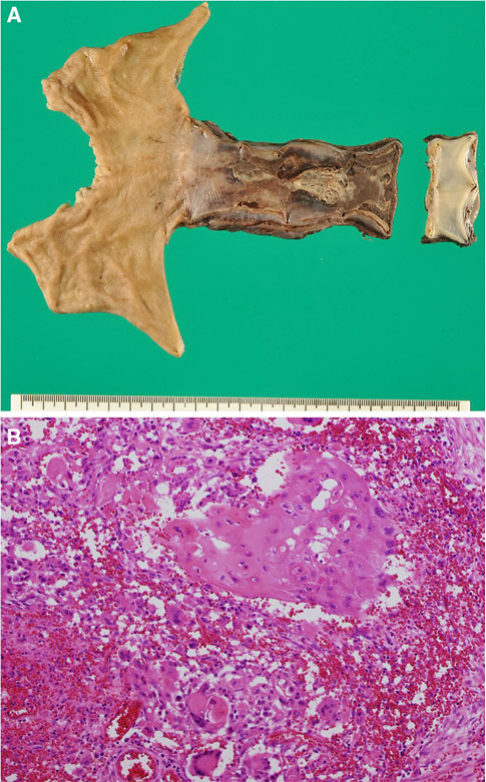
**a**, **b** Macroscopic findings of the resected specimen showed marked erosion and active ulcer (**a**). Histological findings for surgical specimen (**b** ×100). Almost all cancer cells became necrotic, and no viable cancer cells existed

The patient had spike fever with leukocytosis [white blood cells (WBCs) 19,020/μl]. Serum G-CSF level was also increased (53.7 pg/ml; normal range, <18.1 pg/ml). Immunohistochemistry revealed positive staining for anti-G-CSF antibody in the cytoplasm of cancer cells (Fig. 2b). The patient was diagnosed with G-CSF-producing locally advanced esophageal squamous cell carcinoma. The clinical diagnosis was T4: tumor invaded aorta (Fig. 3a), with regional lymph node metastases (N1), according to the Japanese Classification of Esophageal Cancer, tenth edition [10, 11]. Only 5-fluorouracil administration (800 mg/m^2^ body surface area, days 1–5, 2 courses) and localized radiation as neoadjuvant therapy were designed because of transplantation-induced chronic renal failure, estimated creatinine clearance rate using Cockcroft–Gault formula of 39.1 ml/min, and irradiation history for leukemia. A total dose of 41.4 Gy was given in 23 fractions of 1.8 Gy, with 5 fractions per week starting on the first day of the first cycle of chemotherapy. The target volume comprised the primary tumor and enlarged regional lymph nodes. The spinal cord was excluded from the target area as much as possible, so as to not extend beyond 15 Gy. The patient completed the neoadjuvant therapy without severe adverse events, and the preoperative therapy evaluation was partial response (PR) with downstaging from T4 to T3 (Fig. 3b). The patient underwent transthoracic esophagectomy with three-field lymph node dissection and gastric tube reconstruction. There were marked erosion and active ulcer with necrotic or degenerated tumor cells in the resected esophageal specimen, showing pathological complete response (pCR) (Fig. 4). Tumor cells were substituted with degenerate keratotic lesion surrounding foreign-body giant cells. There were no viable cancer cells. Hence, the final stage was CRT-pT0N0M0, CRT-Grade3. After the operation, neutrophils and serum G-CSF decreased to normal levels (Table 1). The patient had recurrence of regional and distant multiple lymph node metastases at 3 months after operation.

**Table 1:** Changes in examination data before and after treatment

	Preoperative		Postoperative
Leukocytes (/μl)	19020		6130
Neutrophils (/μl)	14018		3568
G-CSF (pg/ml)	53.7	→	21.4
IL-6 (pg/ml)	13.7		Untested
SCC (ng/ml)	4.4		2.1

*G-CSF* granulocyte colony-stimulating factor, *IL* interleukin, *SCC* squamous cell carcinoma-related antigen

## Discussion

Advances in cancer treatment mean that today almost 80 % of children diagnosed with cancer are alive at least 5 years after diagnosis. More than fifty thousand childhood cancer survivors are alive in Japan. Assessing the risk of second and subsequent primary cancer is ever more important. In some large cohorts, the overall risk of second cancer development rose about 4- to 6-fold over what was expected [1–4]. Risk of subsequent cancer was increased by treatment with dose-relative radiation therapy exposure and increased with age (i.e., 15–21 years) at primary childhood cancer diagnosis [1]. The standardized incidence ratio for digestive subsequent primary cancer was 4.6 times more than expected [3]. Moreover, patients treated with chemotherapy and radiotherapy followed by hematopoietic cell transplantation have an increased risk of developing second primary malignancies [12–14]. Majhail et al. [15] reported that significant elevated risks for esophageal cancer were observed in allogeneic hematopoietic cell transplant recipients who had received conditioning without total-body irradiation. For acute lymphoblastic leukemia, craniospinal irradiation (CSI) was performed to prevent or treat central nervous system leukemia [16–19]. In the present case, the patient had history of multimodal therapy for leukemia without evidence for typical risk factors for developing esophageal squamous cell carcinoma (abuse of nicotine or alcohol). The primary esophageal cancer occurred within the irradiated area after a long latent interval between irradiation and development of cancer. Therefore, we diagnosed this case as second and subsequent primary esophageal squamous cell carcinoma.

Since the 1960s, case reports of radiation-induced esophageal second cancer have been published across the world. Micke et al. [20] reported 66 cases with radiation-induced esophageal cancer in their review. The median latency from irradiation to diagnosis of second cancer was 15 years (range 2–63 years), the median dose of irradiation was 40 Gy (range 18.6–68 Gy), and the histological type in most cases was squamous cell carcinoma, which fits our case. There are also several reports of increased risk of esophageal cancer after adjuvant radiation therapy for primary breast cancer [21–23]. Thus, it is important for patients who have been treated for cancer by radiotherapy, chemotherapy, and allogeneic hematopoietic cell transplantation to be checked for second cancer occurrence, particularly within the irradiated area.

To our knowledge, the present case is the first report of G-CSF-producing second primary esophageal squamous cell carcinoma. Fahey [24] reported unusual leukocyte responses in primary cancer of the lung, and Robinson [5] confirmed increased G-CSF level in malignancy patients’ serum and urine in 1974. Since then, G-CSF-producing cancer has been reported in various malignancies (i.e., lung [6], stomach [7], liver [8], gallbladder [9], etc.). In cases of esophageal cancer, especially squamous cell carcinoma, production of G-CSF is rare.

Our case fits the criteria for a diagnosis of G-CSF-producing tumor: (1) marked leukocytosis, (2) elevation of serum G-CSF concentration, (3) decrease in leukocyte count after removal of the tumor, and (4) positive staining for G-CSF in tumor cells. There have been several case reports on G-CSF-producing esophageal squamous cell carcinoma [25–31], but to date none with history of irradiation (Table 2). We could not examine the surgical specimen in detail by immunostaining because of pCR. It is certain that multiple factors are associated with second primary carcinogenesis. However, it is impossible to prove whether treatment of leukemia, including radiotherapy, chemotherapy, and bone marrow transplantation, plays a causal role in the development of G-CSF-producing tumor.

**Table 2:** Characteristics of G-CSF-producing esophageal squamous cell carcinoma

Case	Author	Age (years)	Gender	Leukocyte count (/μl)	Neutrophil sequestration (%)	G-CSF level (pg/ml)	Past history	Degree of differentiation	Treatment	Outcome	
1	Watanabe	81	F	22100	91	1175	NP	NM	BSC	0.5 months	Died
2	Matsumoto	66	M	42500	92	154	NP	Moderately	Op, adjuvant CRT	1 year 4 months	Died
3	Ichiishi	66	M	33900	97	180	NM	Moderately–poorly	BSC	2 months	Died
4	Komatsu	73	M	45700	92	231	NM	Moderately	Op	19 months	Alive
5	Nakata	56	M	24300	83	78	NM	Moderately	Op, adjuvant CRT	16 months	Alive
6	Mimatsu	69	M	19600	81	113	Hypertension	Poorly	Radiation	7 months	Died
7	Tanabe	76	M	24600	NM	134	NM	Moderately	Op, palliative CRT	10 months	Died
8	Our case	30	M	19020	74	53.7	Leukemia	Well	Neoadjuvant CRT, Op	3 months	Recurrence

*G-CSF* granulocyte colony-stimulating factor, *NP* nothing particular, *NM* not mentioned, *BSC* best supportive care, *CRT* chemoradiotherapy, *Op* operation

Although there have been a number of reports on incidence of therapy-induced second solid tumors, few have focused on treatment, and prognosis is considered to be poor [32, 33]. Impairment of bone marrow and other organ function limits treatment intensity. Patients receiving immunosuppressive therapy for organ transplants are prone to development of cancer. Additionally, a G-CSF-producing tumor indicates aggressive growth with poor prognosis. In fact, most patients with G-CSF-producing esophageal squamous cell carcinoma die within only 1 year in spite of multimodality therapy. Because G-CSF-producing esophageal squamous cell carcinoma is extremely rare, the optimal treatment strategy is unclear at present. In our case, it was impossible to perform definitive surgery due to locally advanced disease (T4) at diagnosis and impossible to administer definitive chemoradiotherapy due to medical history of irradiation and chronic renal failure. Recently it has become possible to irradiate the target lesion with minimal exposure to the surrounding organ because of advances in irradiation technique. Therefore, we selected neoadjuvant chemoradiation consisting of only 5-fluorouracil and localized radiation. Surgery after irradiation is far more challenging compared with primary surgery because the risk of operative morbidity and mortality increases. To maintain microvascular blood flow, we preserved the tissue surrounding the trachea. This should be achieved using conservative nodal dissection rather than radical dissection in the irradiated field. We dissected the minimal area of the enlarged lymph nodes before CRT. While R0 resection could be performed following neoadjuvant therapy, achieving good clinical response and pCR, the patient relapsed promptly after surgery. Although it is controversial whether chemotherapy and radiotherapy are useful or not for G-CSF-producing cancers, multimodal intensive therapy based on that for conventional, non-G-CSF-producing, esophageal cancer might be recommended at least for squamous cell carcinoma.

In conclusion, G-CSF-producing esophageal squamous cell carcinoma is relatively rare and denotes a dismal prognosis. In the case of a patient with leukocytosis, the possibility of G-CSF-producing cancer should be considered. It is important for patients who have history of treatment for cancer by radiotherapy, chemotherapy, and allogeneic hematopoietic cell transplantation to be checked for second cancer occurrence, particularly within the irradiated area.
